# Cockroach Infestation in Bahir Dar Area of Ethiopia: A Transition Between the Savanna Biome and the Middle Afromontane Hotspot

**DOI:** 10.1155/jotm/9935228

**Published:** 2026-01-07

**Authors:** Yelfwagash Asmare, Melaku Wale, Sileshi Minbale

**Affiliations:** ^1^ Department of Biology, Bahir Dar University, Bahir Dar, Ethiopia, bdu.edu.et

**Keywords:** cockroaches species, habitats, household settings, management practices

## Abstract

**Background:**

In urban areas of developing countries, cockroaches pose a significant public health threat by spreading diseases and contaminating food sources. This study aimed to assess the infestation levels of indoor cockroach species across various household settings and to evaluate community management practices.

**Methods:**

The study was conducted from April to June 2022, utilizing sticky traps placed within 2 × 2 m distance transects. Morphological identification of cockroach species was performed using standard taxonomic keys. Data on community management practices were gathered through a well‐structured questionnaire. A factorial ANOVA was conducted using SAS statistical software to determine significant differences in cockroach abundance based on the various factors such as sampling months, habitats, species, and sexes. Descriptive statistics were applied to analyze questionnaire data.

**Results:**

A total of 2670 cockroach individuals were collected. The overall ANOVA indicated a statistically significant difference in cockroach abundance among the measured variables and factors (*F* = 11.7, DF = 59, 540, *p* < 0.0001). A significant interaction was also observed between month, habitat, and cockroach sexes (excluding species) (*F* = 3.1, DF = 8, *p* < 0.0022). The interaction between cockroach species and habitats was significant (*F* = 4, DF = 33.44, *p* < 0.0001), while interactions between species and months (*F* = 2, DF = 1.88, *p* = 0.15) and species and sexes were not significant (*F* = 1, DF = 0.87, *p* = 0.35).

**Conclusion:**

Grain mills with *Periplaneta americana* and residential areas with *Blattella germanica* exhibited higher infestation levels compared to other habitats. Households primarily relied on chemical and physical methods for cockroach control. Further studies should be conducted on a broader scale to enhance understanding and management of cockroach infestations.

## 1. Introduction

Cockroaches are an ancient group of arthropods representing one of the most successful life forms. They have high adaptability to a wide range of habitats and environmental conditions from Arctic cold to tropical heat [1] which existed for more than 350–400 million years on earth [2]. There are approximately 4600 species of cockroaches worldwide, among which about 50 species often coexist with human populations, and few are considered indoor health pests [3–5]. The majority of cockroach species are not domiciliary pests; instead, they are living among decaying leaves, under tree bark, under stones, or in soil, and this large and untapped resource is likely to provide a bountiful source of microbial diversity [6].

Though cockroaches are found in all habitats, their origin is referred to as North Tropical Africa [7]. Recently, cockroach infestations have been increasing across the world [6]. Commonly known pest cockroach species are the German cockroach (*Blattella germanica*), the American cockroach (*Periplaneta americana*), the brown‐banded cockroach (*Supella longipalpa*), and the oriental cockroach (*Blatta orientalis*) [6]. Of the aforementioned species, German cockroaches are the most well‐known medically important urban pests found in houses and restaurants [8, 9]. The presence of cockroach populations in and around urban areas is an indication that food, moisture, and harborage resources are present in the vicinity. These conditions allow them to proliferate and explode into a large cockroach population [10].

Cockroaches are mechanical vectors which transmit disease‐causing pathogens that may potentially contaminate food, kitchen utensils, and food preparation surfaces and produce bad odors [11–13]. Cockroaches are considered as risk factors and health hazards to human beings [14, 15]. Especially German cockroaches have the filthy ability to spoil food and produce psychological distress [16]. Studies carried out in hospitals and restaurants of Ethiopia revealed that the pathogenic bacteria are found on the body surface of cockroaches [17].

Research efforts by various institutions have increasingly focused on understanding the role of cockroaches as transmitters of pathogens, highlighting their public health implications. For example, an effort was made to compare the fungal contamination of cockroaches in clinical and nonclinical settings, revealing noteworthy findings in Iran [18]. While hospitals exhibited the highest overall infestation of cockroaches, households demonstrated a greater percentage of fungal contamination on these pests. This indicates that cockroaches in residential areas are more likely to carry fungi, including opportunistic pathogens such as *Aspergillus*, *Candida*, and *Rhizopus*. Notable results of the study included a significant contamination of *Blattella germanica* and the identification of medically relevant fungi, underscoring the potential health risks posed by cockroaches as disease vectors.

A systematic review and meta‐analysis of the infestation of cockroaches in the human dwelling environments gave a good picture of their occurrence [7]. Cockroach infestations in homes were found to be widespread, driven by factors such as food, water, and shelter, causing allergies, asthma, and potential disease spread (like dysentery and typhoid) through their feces and body parts, leading to health issues like asthma flare‐ups. Control involves strict sanitation (sealed food/trash and clean surfaces), eliminating moisture (fixing leaks), sealing entry points (cracks and holes), reducing rubbish, and using baits/insecticides, with German cockroaches being a major pest [7].

Other systematic reviews and meta‐analysis efforts confirmed that cockroaches are significant vectors for medically important fungi, carrying numerous species (like *Aspergillus*, *Candida*, and *Rhizopus*) that cause opportunistic infections, especially in immunocompromised individuals, highlighting their role in spreading pathogens in hospitals and homes, with contamination rates varying by location and cockroach species [19]. Furthermore, cockroaches are found to be significant vectors for medically important bacteria, mechanically spreading pathogens like *E. coli*, *Staphylococcus aureus*, *Salmonella*, and *Klebsiella* from unsanitary environments to food and surfaces, contributing to foodborne and hospital‐acquired infections, and often carrying multidrug resistant strains that pose serious public health hazards. They pick up bacteria on their bodies and in their digestive tracts, excreting them through feces and regurgitation, contaminating food, utensils, and hospital equipment, thereby facilitating disease transmission to humans [20]. The feeding habits and morphology of cockroaches make them disease‐transmitting arthropods. Therefore, studies on factors which help cockroaches to proliferate and factors which can suppress their population are useful to manage our living and working settings. The objective of this study was to determine infestation level and species composition of cockroaches at different settings/habitats including residential, hotel, hospital, students’ dormitory, and mill houses in the study area. The habitat characteristics and environmental factors in relation to the population pattern of cockroaches are discussed.

## 2. Materials and Methods

### 2.1. Description of the Study Area

Bahir Dar is a city located in northwest Ethiopia with a tropical savanna climate, a transition between the Ethiopian highlands and the savanna grassland biome. It is located 565 km to the northwest of Addis Ababa and has a latitude of 11° 59′ N, a longitude of 37° 39′ E, and an average altitude of 1840 m (5970 ft.) above mean sea level. The average annual temperature is 20.1°C, and the rainfall averages 1839 mm [21]. The rainy period lasts from May up to October. The maximum rainfall occurs during the summer season from June to August, whereas the short rainfall takes place in the spring season from September to October [21, 22]. The rainy season accounts for nearly over 96% of the total annual rainfall [21, 22]. Bahir Dar, the capital of the Amhara region in Ethiopia, is a rapidly growing secondary city in the Amhara Region, Ethiopia, and it is one of the leading tourist destinations in Ethiopia, with a variety of attractions in the nearby Lake Tana and Blue Nile River [23]. Cockroaches have implications to the tourism industry, which was otherwise improving steadily [24].

### 2.2. Cockroach Sampling and Species Identification

A cross‐sectional study design was applied to determine infestation level and species composition of cockroaches at different household settings. The study was conducted from April 2022 to June 2022, which corresponded to the warmest season of the year. Cockroaches were collected from different household settings such as residential houses, mill houses, hospitals, hotels, and student dormitories (10 replications each). The sites of each habitat were selected randomly in each study month. Simple sticky traps were made from locally available materials for collecting cockroaches. Petri dishes (100 mm diameter), their interior painted with 25 mg Vaseline for getting glue with 10 g bananas as bait. Traps were placed on the floor within a 2 × 2 m transect.

Four traps arranged with similar distances between them within transect was the setup of cockroaches sampling in the study settings and each was observed to collect the samples after 24 h. Adult cockroaches collected from the four traps at transect setup of a given replicates of a setting type for a month were pooled in a labeled jar. Every study month adult cockroaches collected from 10 replicates of each the five settings were placed in 10 separate labeled jars. Ten jars of each five settings totally 50 jars with sample cockroaches for every month was transported to laboratory for identification of sexes and species within 24 h of collection. Morphological identification of the cockroach species was carried out using standard taxonomic keys, dichotomous keys and stereomicroscopes [24]. The data were recorded in data sheet for statistical analysis.

### 2.3. Sample Size Determination and Sampling Technique on Cockroach Management Practices

The sample size of resident households to study their knowledge, attitude, and practice on cockroach management was determined by using the formula [25]:
(1)

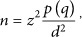


where *n* = minimum sample size of households. *Z* = statistics for the level of confidence of 95% = 1.96 (error 5% = 1.96). *p* = expected infestation level of cockroaches for 0.5. *d* = precision or small error of estimate 0.05 (statistical significance set). *q* = 1 − *p* = the proportion in the sample population not expected to be infested.

The sample size determined by the formula is about 320. The lottery system was applied to generate the sample households. The researcher explained the main purpose of the study and got permission from the respondents. For each of the selected a household, a consenting adult respondent was given a well‐structured questionnaire to fill in.

### 2.4. Data Analysis

A factorial ANOVA was invoked using SAS statistical software to determine whether there was a significant difference in cockroach abundance with respect to sampling months, habitats, species and their sexes, and interactions of all combinations. Use of various methods of pest control (chemical, physical, and biological) by residents was analyzed using chi‐square analysis. Users were categorized into frequent, rare, and nonusers.

## 3. Results

A total of 2670 cockroach specimens belonging to two species, *B. germanica* (1536) and *P. americana* (1134 individuals), were found during the entire study period. Numbers varied according to sampling habitat, month, species, or sexes. Figure 1 shows the two species found during the study.

Figure 1Picture of two cockroach species: the German cockroach (a) and the American cockroach (b) identified in the Bahir Dar area. (Photo: Yelfwagash Asmare, Bahir Dar University, Ethiopia).

**Figure:**
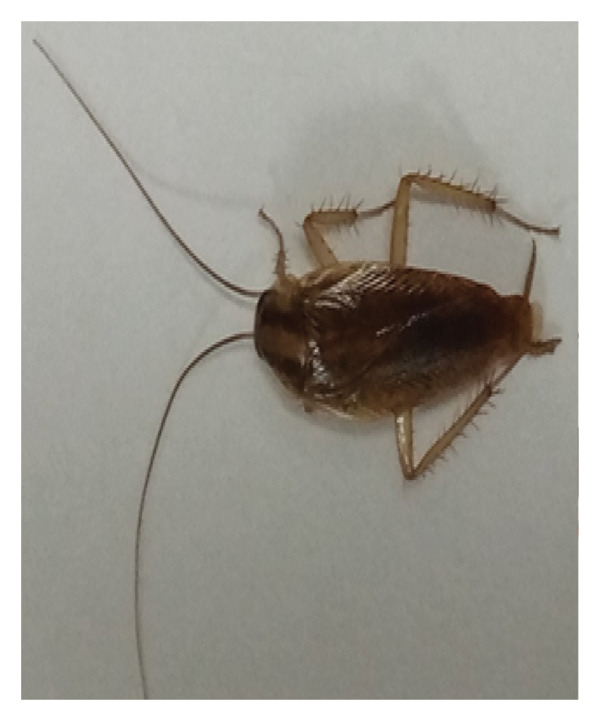
(a)

**Figure:**
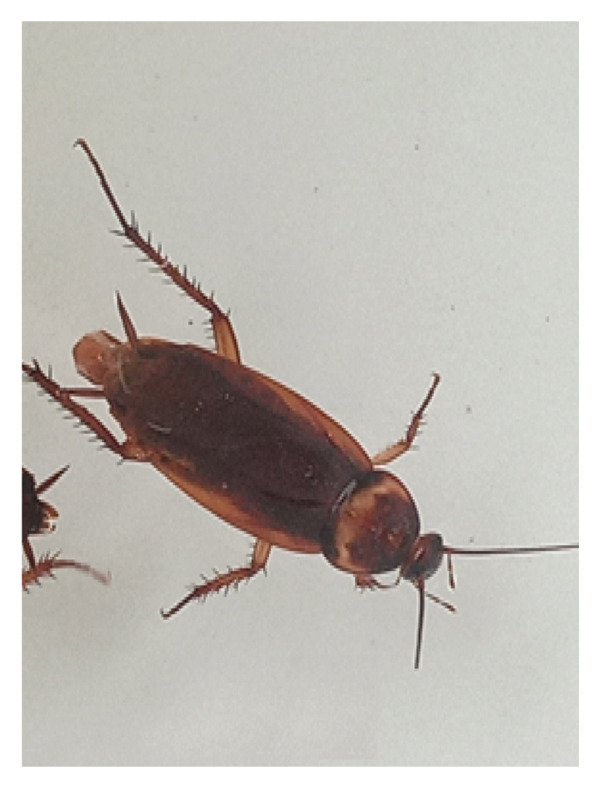
(b)

### 3.1. Interaction Between Factors

The overall ANOVA for differences in cockroach abundance was statistically significant (*F* = 11.7, DF = 59.540, *p* < 0.0001). While the number of cockroaches was significantly different between the main factor levels, i.e., levels of sampling months, habitats, species, and sexes, the interactions were not significant for higher‐order interactions, e.g., Month ∗ Habitat ∗ Species ∗ Sex (Table 1). One high‐order significant interaction was Month ∗ Habitat ∗ Sex (excluding species) (*F* = 3.1, DF = 8, *p* < 0.0022). The higher the order of interaction, the less likely it turned significant.

**Table 1:** Analysis of variance output for the various factorial combinations.

Source (factor)	Degrees of freedom	Sum of squares	*F* ratio	Prob > *F*
Month	2	203.13	7.92	0.0004
Habitat	4	3967.75	77.37	< 0.0001
Month ∗ Habitat	8	755.77	7.37	< 0.0001
Species	1	269.34	21.01	< 0.0001
Month ∗ Species	2	48.19	1.88	0.1537
Habitat ∗ Species	4	1714.81	33.44	< 0.0001
Month ∗ Habitat ∗ Species	8	122.61	1.20	0.2994
Sex	1	897.93	70.04	< 0.0001
Month ∗ Sex	2	84.80	3.31	0.0374
Habitat ∗ Sex	4	292.36	5.70	0.0002
Month ∗ Habitat ∗ Sex	8	314.66	3.07	0.0022
Species ∗ Sex	1	11.21	0.87	0.3502
Month ∗ Species ∗ Sex	2	16.42	0.64	0.5274
Habitat ∗ Species ∗ Sex	4	72.91	1.42	0.2254
Month ∗ Habitat ∗ Species ∗ Sex	8	55.61	0.54	0.8248

### 3.2. Population Fluctuation Pattern of Different Sexes of Cockroaches at Different Habitats and Months

Generally, the number of males was more than females especially in residential and grain milling habitats. Numbers were higher in April than in other months, and their numbers generally declined as we move from residential to grain mill, to hospital, to hotel, and student dorms. Student dormitories had the lowest and residential areas the highest cockroach numbers (Table 2).

**Table 2:** Mean number of cockroaches at various levels of sampling months, habitats, and sexes.

	April		May		June	
	Male	Female	Male	Female	Male	Female
Residential	11.25ab	6.75c–e	5.1d–h	4.8d–h	11.9a	6.45c–e
Grain mill	11.2ab	5.5c–f	9.7a–c	4.5e–h	5.15d–g	3.6e–h
Hospital	7.0b–e	3.45e–h	3.45e–h	3.0e–h	9.0a–d	3.4e–h
Hotel	2.0f–h	1.0g–h	2.1f–h	1.7f–h	1.95f–h	1.35f–h
Student dorm	1.95f–h	0.85h	1.6f–h	0.85h	1.75f–h	0.95g–h

*Note:* Levels not connected by same letter are significantly different.

### 3.3. Cockroach Species Found in the Study Area

Despite the expectation, we found only two cockroach species in the study area. Interaction between cockroach species and habitats was significant (*F* = 4, DF = 33.44, *p* < 0.0001) but not between species and months (*F* = 2, DF = 1.88, *p* = 0.15) or species and sexes (*F* = 1, DF = 0.87, *p* = 0.35) (Table 1). Generally, the mean number of *B. germanica* per trap (5.1 ± 0.2) was significantly higher than *P. americana* (3.8 ± 0.2).

### 3.4. Abundance of Cockroach Species at Different Habitats

The two species were most abundant in a habitat of their own, whereby *B. germanica* was numerous in residential areas and *P. americana* in grain mills (Table 3). That means in residential areas, one would find more *B. germanica* and in grain mills, more *P. americana*. Other habitats were less preferred. That was why the interaction effect between habitat and species was significant.

**Table 3:** Mean number of cockroaches of different species at different habitats.

	*B. germanica*	*P. americana*
Residential	10.67a	4.75b
Grain mill	4.38bc	8.91a
Hospital	5.56b	4.2bc
Hotel	2.33cd	1.03de
Student dorm	2.65cd	0.0e

*Note:* Levels not connected by same letter are significantly different.

### 3.5. Variation in Different Habitats and Months

**Figure 2:**
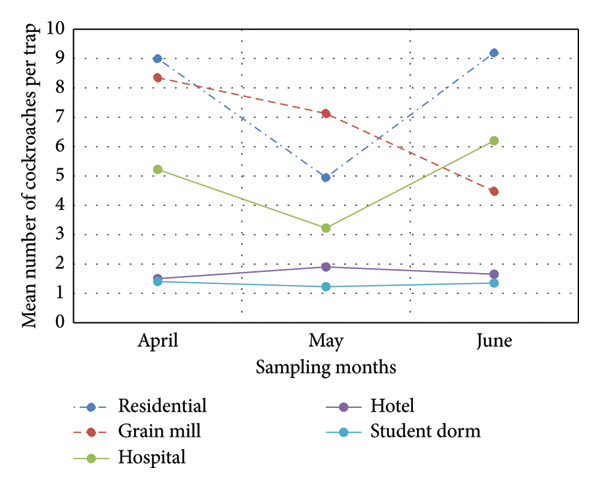
Variation of cockroach numbers in different habitats and sampling months (during the hottest months of the year—April to June).

Grain mills and residential areas were found more infested than other habitats (Figure 2). Cockroach numbers were less in May than in April and June. Numbers were the lowest in student dormitories and hotels.

### 3.6. Residents’ Actions Taken to Control Cockroaches

Residents used chemical, physical, and biological but not cultural control methods against cockroaches (Table 4). Frequent users ranged between 1.5% and 23.4%; those who did not practice any control method at all ranged between 50% (chemical) and 96% (biological). However, the majority of them did not practice any control method. None of them used cultural practices.

**Table 4:** Cockroach control practices by residents.

Variable	Category	Frequency (# respondents)	Percentage	Chi‐square test
Chemical	Frequently	75	23.4	*χ* ^2^ = 391, *p* < 0.0001
	Rarely	86	26.8	
	Not used	159	49.6	

Physical	Frequently	29	9.1	*χ* ^2^ = 399.3, *p* < 0.0001
	Rarely	16	5	
	Not used	275	82	

Biological	Frequently	5	1.5	*χ* ^2^ = 558.8, *p* < 0.0001
	Rarely	9	2.8	
	Not used	306	95.6	

## 4. Discussion

This study has underpinned the fact that residential areas were found to be the most infested habitats by cockroaches. This shows the poor level of keeping residential areas clean and tidy, which might have been caused by the low standard of living of the community in general. Student dormitories are regularly sprayed with pesticides, and cockroaches might not have a foothold there (pers. obs.). While the cleanliness of hospitals and hotels is better maintained, residential, and grain mills are not. Grain mills always look filthy because of the constant deposition of leftover flour. This study also elucidates that *P. americana* was the dominant species in grain mills but not in others. On the other hand, *B. germanica* was dominant in residential areas. This situation tells us that species serve as habitat indicators.

Cockroaches infest apartments, homes, food handling establishments, hospitals, and healthcare facilities worldwide [3–5, 10]. The German cockroach, an indoor species, exploits conditions associated with high‐density human settlements and impoverished living conditions. One can wonder how only two cockroach species were found in the current study, given that over 3500 cockroach species exist worldwide [26] and the German cockroach originates between Sudan and Ethiopia [27]. Although it is said that the German cockroach originates in the Sudan‐Ethiopia border, neither this species nor any other cockroach species is found in the entire Ethiopian highlands, i.e., above 2500 m (8202 feet) above sea level (m.a.s.l.) (pers. obs.). The second author of the current paper is from such areas, and he had seen cockroaches and heard about them for the first time only after he traveled to other parts of the country, i.e., to the lowlands, as a civil servant. However, cockroaches have managed to be seen at intermediate altitudes (about 2000 m.a.s.l.) recently because of climate change and modern warm residential houses. Recent molecular studies suggest that they originate in India or Myanmar [28]. According to fossil records, they have been around for about 300 million years [29].

Of the thousands of species, a few are economically important including the American cockroach, Australian cockroach, oriental cockroach, brown‐banded cockroach, and German cockroach [27]. Two of these were found in the present study area.

In the current study, *B. germanica* had a slight edge advantage in number (58% of total) than *P. americana* (42%). This is unlike other countries such as South Africa, where the majority belonged to the German cockroach, *B. germanica* [30]. The German cockroach is found throughout the world along with human habitats [9, 10, 27, 31]. It does not survive in locations away from humans or human activity. The major factor limiting German cockroach survival appears to be cold temperatures. The German cockroach is the cockroach of real concern, the species that gives all other cockroach species a bad name. It plagues multifamily dwellings as far as the temperature is ideal for its reproduction [27, 31]. German cockroaches spoil food with their feces and defensive secretions, physically transport or harbor pathogens, may cause severe allergic reactions, and in extremely heavy infestations may bite humans and feed on food residues on the faces of sleeping persons [27, 31, 32]. In addition, some scientists suggest that German cockroach infestations may cause human psychological stress and that the stigma associated with infestations alters human behavior to the detriment of healthy social interaction.

On the other hand, other people consider the German cockroach an aesthetic pest rather than a medical or otherwise, the action threshold for this insect depends upon the tolerance of the residents living in infested dwellings. Most people associate cockroach infestations with poor sanitary conditions, and they want them eradicated from their dwellings by whatever means necessary. In fact, cockroaches can cause food poisoning and asthma (allergic reactions) [27, 31]. Cockroaches and mites are increasingly considered as sources of asthmatic reactions in crowded and impoverished urban areas [10]. Therefore, cockroaches are no longer of only aesthetic importance but also of health. Further, according to a study done in hospitals and restaurants in Addis Ababa, Ethiopia, German cockroaches were found to be a reservoir and potential vector of some food‐borne pathogens and may cause multiple drug resistance in hospitals and food catering establishments [32]. Cockroaches carry organisms that cause diarrhea, dysentery, cholera, leprosy, plague, typhoid fever, and viral diseases such as poliomyelitis [31, 32].

The traps we used in the current study were sticky traps with bananas as bait, and this might have implications in the number of cockroaches trapped. For example, various traps, i.e., bread with beer, peanut butter, Trapper roach attractant, NAF430 gel bait, and invite lure in a choice test, were investigated for their efficiency as cockroach traps. In this case, attractants with baits increased the number of cockroaches trapped in sticky traps compared with an unbaited traps, whereby bread with beer was by far the most attractive bait, increasing trap catches by 34‐fold over the unbaited control [27, 33, 31, 32].

Researchers have shown that cockroaches produce aggregation pheromones—like the volatile sex pheromone blattellaquinone—to attract male cockroaches, and this has the potential to be used in pest‐control programs by aggregating nymphs and arresting their activity on fecal deposits [10, 11, 31, 33]. This helps minimize the use of synthetic pesticides and thereby the likelihood of insecticide resistance and human exposure to insecticides. Poor sanitary practices, failure to repair structures, and messy living premises contribute to large populations of cockroaches.

The current study was conducted during the warmest time of the year in the study area, Bahir Dar, Ethiopia, assuming that numbers get higher during this time. Similarly, a study conducted in Egypt showed that German cockroach populations varied with respect to months of the year, rural or urban areas, and kitchen or other indoor shelters [31, 32]. Eggs fail to hatch when temperatures are < 15°C [10, 31]. So, the most suitable reproductive season in the current study area was captured. That means, assuming cockroach reproduction basically depends on temperature, the likelihood of more reproduction than the current report is unlikely because the rest of the year, the temperatures are low.

## 5. Conclusion

In this study we have confirmed that at least two species of cockroaches exist in the different indoor habitats of the study area, northwestern Ethiopia, a borderline or transition between the Ethiopian portion of the Afromontane and the African savanna biome. Populations varied temporally and spatially, i.e., more *B. germanica* in residential areas and more *P. americana* in grain mills and less in May than in other months. Interactions were also observed between some of the factors involved (sources of variation) but not in others. The highest possible rate of reproduction was between April and June, and that means more reproduction than the current study is unlikely, assuming the role of high temperature for cockroach reproduction.

## Ethics Statement

The authors have nothing to report.

## Conflicts of Interest

The authors declare no conflicts of interest.

## Author Contributions

Yelfwagash Asmare conceived the original idea, drafted the proposal, formulated the data collection and analysis plan, and prepared the initial manuscript. Melaku Wale reviewed the draft proposal, made amendments, provided technical assistance during data collection, organized and analyzed the data, and restructured the manuscript. Sileshi Minbale authored the initial proposal, defended it, incorporated feedback, conducted field and laboratory work, collected data, participated in data analysis, wrote the final technical report, and contributed to the manuscript preparation.

## Funding

This study was financially supported by a grant from Bahir Dar University.

## Data Availability

Data are available from the corresponding author upon request.

## References

[1] Chamavit P. , Sahaisook P. , and Niamnuy N. , The Majority of Cockroaches From the Samutprakarn Province of Thailand are Carriers of Parasitic Organisms, Excli Journal Experimental and Clinical Sciences. (2011) 10, 218–222. PMC5109011 27857676

[2] Cochran D. G. , Cockroaches’ Biology, Distribution and Control, World Health Organization. (1999) 99, 1–83.

[3] Tinker K. A. and Ottesen E. A. , The Core Gut Microbiome of the American Cockroach, *Periplaneta americana*, is Stable and Resilient to Dietary Shifts, Applied and Environmental Microbiology. (2016) 82, no. 22, 6603–6610, 10.1128/aem.01837-16, 2-s2.0-84995468835. 27590811 PMC5086554

[4] Richards C. , Otani S. , Mikaelyan A. , and Poulsen M. , *Pycnoscelus surinamensis* Cockroach Gut Microbiota Respond Consistently to a Fungal Diet Without Mirroring Those of Fungus-Farming Termites, PLoS One. (2017) 12, no. 10, 10.1371/journal.pone.0185745, 2-s2.0-85030467059. PMC5626473 28973021

[5] Lampert N. , Mikaelyan A. , and Brune A. , Diet Is Not the Primary Driver of Bacterial Community Structure in the Gut of Litter-Feeding Cockroaches, Microbiology. (2019) 19, no. 1, 10.1186/s12866-019-1601-9. PMC6864750 31666028

[6] Nasirian H. , Rapid Elimination of German Cockroach *Blattella germanica*, by Fipronil and Imidacloprid Gel Baits, Iranian Journal of Arthropod-Borne Diseases. (2008) 2, 37–43.

[7] Nasirian H. , Infestation of Cockroaches (Insecta: Blattaria) in the Human Dwelling Environments: A Systematic Review and Meta-Analysis, Acta Tropica. (2017) 167, 86–98, 10.1016/j.actatropica.2016.12.019, 2-s2.0-85007492378. 28012906

[8] Bayer B. , Palatability and Efficacy of Emamectin Benzoate Gel Bait on Four Pest Cockroach Species, 2007, University of Florida, Gainesville, M.Sc. thesis.

[9] Snoddy T. , Distribution and Population Dynamics of the Asian Cockroach (Blattella Asthenia Mizukubo) in Southern Alabama and Georgia, 2007, Auburn University, Auburn, M.Sc. thesis.

[10] Bonnefoy X. , Kampen H. , and Sweeney K. , Public Health Significance of Urban Pests, 2008, 8, World Health Organization.

[11] Shahraki G. H. , Parhizkar S. , and Nejad A. R. S. , Cockroach Infestation and Factors Affecting the Estimation of Cockroach Population in Urban Communities, International Journal of Zoology. (2013) 2013.

[12] Adenusi A. , Akinyemi M. , and Akinsanya D. , Domiciliary Cockroaches as Carriers of Human Intestinal Parasites in Lagos Metropolis, Southwest Nigeria: Implications for Public Health, Journal of Arthropod Borne Diseases. (2018) 12, no. 2, 141–151, 10.18502/jad.v12i2.40. 30123808 PMC6091797

[13] Donkor E. , Cockroaches and Food-Borne Pathogens, Environmental Health Insights. (2020) 14, 1–6, 10.1177/1178630220913365. PMC7218330 32425541

[14] Alam M. , Awan Z. , Khan M. , Shah A. , Bangash H. , and Rehaman A. , Detection and Isolation of Zoonotic Parasites From American Cockroaches in 48 Households, Pakhtunkhwa Pakistan, International Journal of Advanced Research. (2013) 1, 113–118.

[15] Etim S. , Akpan P. , Vepong G. , Oku E. , and Okon O. , Prevalence of Cockroaches (*Periplanta americana*) in Households in Calabar Public Health Implication, Journal of Public Health and Epidemiology. (2013) 5, 149–152.

[16] Brenner R. , Rust M. , Owens J. , and Reierson D. , Economics and Medical Importance of German Cockroaches, 1995, Oxford University Press.

[17] Kinfu A. and Erko B. , Cockroaches as Human Intestinal Parasite in Two Localities in Ethiopia, Transactions of the Royal Society of Tropical Medicine and Hygiene. (2008) 102, no. 11, 1143–1147, 10.1016/j.trstmh.2008.05.009, 2-s2.0-53349141918. 18579170

[18] Davari B. , Hassanvand A. E. , Nasirian H. , Ghiasian S. A. , Salehzadeh A. , and Nazari M. , Comparison of Cockroach Fungal Contamination in the Clinical and Non-Clinical Environments From Iran, Journal of Entomological and Acarological Research. (2017) 49, no. 2, 10.4081/jear.2017.6758, 2-s2.0-85031800054.

[19] Nasirian H. , Contamination of Cockroaches (Insecta: Blattaria) to Medically Fungi: A Systematic Review and Meta-Analysis, Journal De Mycologie Médicale. (2017) 27, no. 4, 427–4488, 10.1016/j.mycmed.2017.04.012, 2-s2.0-85019138023. 28506564

[20] Nasirian H. , Contamination of Cockroaches (Insecta: Blattaria) by Medically Important Bacteriae: A Systematic Review and Meta-Analysis, Journal of Medical Entomology. (2019) 56, no. 6, 1534–1554, 10.1093/jme/tjz095. 31219601

[21] Amare S. and Kameswara R. , Hydrological Dynamics and Human Impact on Ecosystems of Lake Tana, Norhwestern Ethiopia, Ethiopian Journal of Environmental Studies and Management. (2011) 4, 56–74.

[22] Dechasa F. and Demissie F. , Socio-Economic Impacts of Bahir Dar Tannery: Bahir Dar, Ethiopia, Natural Resource Conservation. (2014) 2, no. 4, 51–58, 10.13189/nrc.2014.020401.

[23] Appelhans N. , Urban Planning and Everyday Urbanization: A Case Study on Bahir Dar, Ethiopia, Urban Study. (2017) 234, 39–99.

[24] Townson H. , Lane R. P. and Crosskey R. W. , Medical Insects and Arachnids, 1993, Cambridge University Press.

[25] Naing L. , Winn T. , and Rusli B. N. , Practical Issues in Calculating the Sample Size for Prevalence Studies, Archives of Orofacial Sciences. (2006) 1, 9–14.

[26] Ogg B. , Ogg C. , and Ferrao D. , Cockroach Control Manual, 2006, 2nd edition, University of Nebraska.

[27] Valles S. , German Cockroach, Blattella germanica (Linnaeus) (Insecta: Blattodea: Blattellidae), 2014, University of Florida.

[28] Tang Q. , Vargo E. L. , Ahmad I. et al., Solving the 250-Year-Old Mystery of the Origin and Global Spread of the German Cockroach, *Blattella germanica* , Evolution. (2017) 121, no. 22, 10.1073/pnas.2401185121. PMC11145273 38768340

[29] India Today Web Desk , Cockroaches Have Existed Since Way Before Dinosaurs, 2018, https://www.indiatoday.in/education-today/gk-current-affairs/story/cockroaches-seem-to-have-existed-way-before-dinosaurs-says-new-study-1170969-2018-02-16.

[30] Molewa M. L. , Barnard T. G. , and Naicker N. , The Prevalence and Distribution of Domiciliary Cockroaches in Rural Areas: A Cross-Sectional Study Design in Limpopo Province, Journal of Epidemiology and Public Health. (2024) 09, no. 2, 145–155, 10.26911/jepublichealth.2024.09.02.02.

[31] Wang C. and Bennett G. W. , Comparison of Cockroach Traps and Attractants for Monitoring German Cockroaches (Dictyoptera: Blattellidae), Environmental Entomology. (2006) 35, no. 3, 765–770, 10.1603/0046-225x-35.3.765, 2-s2.0-33750245603.

[32] Mahmoud M. F. , El-Bahrawy A. F. , El-Sharabasy H. M. , El-Badry Y. S. , and El-Kady G. A. , Ecological Investigation, Density, Infestation Rate and Control Strategy of German Cockroach, *Blattella germanica* (L.) in Two Hospitals in Ismailia, Egypt Arthropod. (2013) 2, 216–224.

[33] Tachbele E. , Erku W. , Gebre-Michael T. , and Ashenafi M. , Cockroach-Associated Food-Borne Bacterial Pathogens From Some Hospitals and Restaurants in Addis Ababa, Ethiopia: Distribution and Antibiograms, Journal of Rural and Tropical Health. (2006) 5, 34–41.

